# Near and mid-infrared optical vortex parametric oscillator based on KTA

**DOI:** 10.1038/s41598-021-86945-1

**Published:** 2021-04-13

**Authors:** Mairihaba Ababaike, Shutong Wang, Palidan Aierken, Takashige Omatsu, Taximaiti Yusufu

**Affiliations:** 1 School of Physics and Electronic Engineering, Xinjiang Normal University, Ürümqi, 830054 Xinjiang China; 2 Laboratory of Novel Light Source and Micro/Nano-Optics, Xinjiang Normal University, Ürümqi, 830054 Xinjiang China; 3 Graduate School of Engineering, Chiba University, 1-33 Yayoi-cho, Inage-ku, Chiba, 263-8522 Japan; 4 Molecular Chirality Research Center, Chiba University, 1-33, Yayoi-cho, Inage-ku, Chiba, 263-8522 Japan

**Keywords:** Solid-state lasers, Nonlinear optics

## Abstract

We investigated high energy, near and mid-infrared optical vortex lasers formed by a 1 μm optical vortex-pumped KTiOAsO_4_ (KTA) optical parametric oscillator. The orbital angular momentum (OAM) of the pump beam can be selectively transferred to the signal or idler output by changing the reflectivity of the output coupler. With this system, 1.535 µm vortex signal output with an energy of 2.04 mJ and 3.468 µm vortex idler output with an energy of 1.75 mJ were obtained with a maximum pump energy of 21 mJ, corresponding to slope efficiencies of 14% and 10%, respectively. The spectral bandwidth (full width at half maximum, FWHM) of the signal and idler vortex outputs were measured to be *Δλ*_*s*_ ~ 1.3 nm (~ 5.5 cm^−1^) and *Δλ*_*i*_ ~ 1.7 nm (~ 1.4 cm^−1^), respectively.

## Introduction

Optical vortices with a helical wavefront carry the orbital angular momentum (OAM), *ℓħ*, for each photon, characterized by an azimuthal phase, exp(*iℓφ*), where *φ* refers to the azimuthal coordinate and *ℓ* is an integer, termed a topological charge, (the number of 2*π* azimuthal phase shifts around the beam axis)^1,2^. The optical vortices with the above mentioned unique properties have been investigated in many applications, including optical manipulation^3^ and trapping^4^, space-division multiplexing telecommunications^5,6^, super-resolution microscopy^7^, and quantum information^8^. In recent years, it has been discovered that the OAM of the optical vortex twists various irradiated materials^9,10^, including metals, semiconductors, and azo-polymers, to fabricate chiral nanostructures. Such chiral nanostructures, which are difficult to fabricate even by utilizing advanced chemical techniques, will potentially open new avenues towards chiral materials science and technology, such as selective identification of the circular dichroism of molecules and chemical composites.

These applications intensely desire the generation of stable and high beam quality optical vortex. Several wavefront elements, such as an azimuthally segmented spiral phase plate (SPP)^11^, diffractive phase holograms^12^, metamaterials^13^, and a *q*-plate^14^, have been employed to produce an optical vortex in the visible and near-infrared region. However, they are typically designed for a specific laser wavelength and inherently constrain the wavelength versatility and OAM controllability of the optical vortex sources.

The nonlinear frequency conversion process^15^ has proven to be the most direct method to extend the wavelength of an optical vortex, including second harmonic generation^16^, sum frequency generation^17^, and stimulated Raman scattering^18^. In particular, the optical parametric oscillator (OPO) has been established as a practical method of coherent radiation from the versatile wavelength (visible ~ midinfrared) and time (continuous wave ~ femtosecond) regions.

Many efforts have been made to generate the optical vortex output in the wavelength regions of 1 μm (with KTP)^19^, 2 μm (with KTP)^20–22^, and 1–3 μm (with LBO)^23^. Further, the 5–18 μm optical vortex generation has been achieved by employing a KTP-OPO in combination with a ZGP difference frequency generator^24^.

The methane, CH_4_, contributes significantly to global warming effects, and it possesses more than 200 absorption lines owing to the ν_3_ vibration mode at 3.2–3.5 μm^25^. Thus, the 3 μm mid-infrared laser sources have been extensively studied to measure the absorption bands of molecules. In fact, the molecular spectroscopy by using the nanosecond mid-infrared OPO system with a linewidth of a few nm has been conducted^26,27^.

A basic framework for super-resolution molecular absorption microscopy with a spatial resolution beyond the diffraction limit by utilizing a vortex pump pulse is explained below^28^. The mid-infrared moderate energy vortex pump beam with a linewidth of a few nm produces a ring-shaped highly-pumped region, in which the absorption of target molecules, owing to selected CH_2_ stretching modes, is highly saturated (minimized or nullified). Thus, the linear absorption of target molecules, confined within a central dark core (its diameter is typically one-tenth the diffraction limit) of the irradiated vortex beam, can be probed by employing the probe Gaussian beam with a further narrower linewidth, for instance, generated from a continuous-wave optical parametric oscillator. Therefore, an optical vortex source in the mid-infrared region (~ 3.4 μm) with a linewidth of a few nm will be potentially applied in the new generation of molecular sciences, such as super-resolution molecular absorption microscopy with a high spatial resolution beyond the diffraction limit, and organic materials processing without the destruction of chemical structures. In fact, several 3 μm continuous wave and femtosecond optical vortex sources have been presented^29,30^. However, high energy 3 μm vortex sources have been not yet established.

Recently, we have demonstrated a milli-joule level tunable optical vortex laser source with a in a near (1.36–1.63 μm) and mid-infrared (3.07–4.81 μm) region, formed of a 1-µm ns vortex pulse-pumped quasi-phase matching MgO-doped periodically poled lithium niobate (MgO: PPLN) optical parametric oscillator^31,32^. However, the extremely large phase matching acceptance bandwidth of the MgO: PPLN crystal results in a broad spectral bandwidth of the output (it was typically measured to be *Δλ*_*i*_ ~ 23 nm, ca. 24.4 cm^−1^)^32,33^.

A KTiAsO_4_ (KTA) crystal possesses high nonlinearity (*d*_*24*_ = 3.43 pm/V), high transmission, and high damage threshold (600 MW/cm^2^)^34,35^ in a wide wavelength region (0.35–5.3 µm), and it also allows the type ΙΙ non-critical phase matching (NCPM), i.e. moderately large acceptance bandwidth without any walk-off effects, among 1 μm pump, 1.5 μm signal and 3.5 μm idler beams. Thus, it would be an excellent candidate for efficient high-energy OPO to generate the near and mid-infrared optical vortex beam.

In this study, we investigate the direct generation of milli-joule level near and mid-infrared optical vortex outputs with a linewidth of < 2 nm by a compact linear cavity OPO, without using any spectral narrowing elements, formed of a KTA crystal. At a maximum pump energy of 21 mJ, vortex signal (1.535 µm) and idler (3.468 µm) vortex outputs with pulse energies of 2.04 mJ and 1.75 mJ were produced, corresponding to slope efficiencies of 14% and 10% for the signal and idler outputs, respectively.

In recent years, second harmonic generation by employing the z-cut uniaxial nonlinear crystal has been performed to achieve spin-orbital angular momentum cascading^36^. It is noteworthy that this configuration impacts significantly phase matching among the pump, signal and idler outputs, thereby yielding an extremely low parametric gain (*d*_*eff*_ ~ 0)^37^.

## Results and discussion

**Figure 1:**
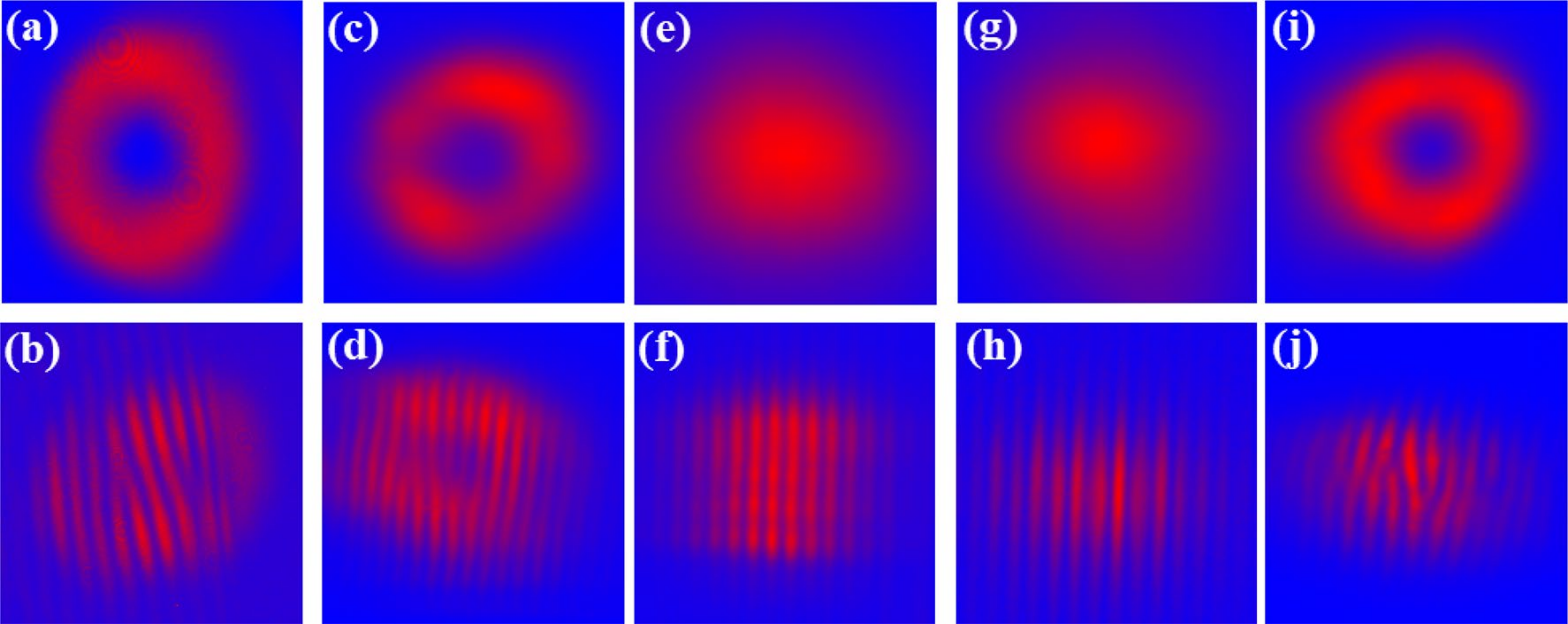
(**a**) Spatial form and (**b**) self-referenced fringes of the pump beam. (**c**) Spatial form and (**d**) self-referenced fringes of the signal output (1.535 µm), (**e**) spatial form and (**f**) self-referenced fringes of the idler output (3.468 µm) obtained in the low-Q cavity configuration. (**g**) Spatial form and (**h**) self-referenced fringes of the signal output (1.535 µm), (**i**) spatial form and (**j**) self-referenced fringe of the idler output (3.468 µm) obtained in the high-Q cavity configuration.

The measured spatial profiles and self-referenced fringes of the pump, signal, and idler outputs are shown in Fig. 1. The pump beam exhibits a vortex mode with an orbital angular momentum of *ℓ* = 1, as depicted in Fig. 1a,b. A plane parallel low-Q cavity with an output coupler (OC, M2) produced the 1.535 µm doughnut-shaped signal output with a first-order phase singularity, as evidenced by a pair of Y-shaped fringes (Fig. 1c,d). The idler output then showed a Gaussian spatial form without any phase singularities (*ℓ* = 0), as evidenced by straight-line fringes (Fig. 1e,f). Note that the handedness of the vortex output is here defined as a right-hand when a pair of upward Y-shaped fringes on the right and downward Y-shaped fringes on the left are observed. Thus, the signal output was assigned to be right-handed and it was identical to that of the pump beam. These results indicate that the OAM of the pump beam was selectively transferred to the signal output.

The OC used in this experiment exhibits high loss (~ 50%) for the signal, and it yields an extremely short buildup time of OPO. The resonating signal output should then carry a beam radius and a helicity of the pump beam, so as to undergo the maximum parametric gain. The OAM conservation law then allows the resulting idler output to exhibit a Gaussian mode profile. In fact, an intracavity photon lifetime τ_*f*_, defined as $\frac{2L}{c}/\left({L}_{in}-\mathrm{ln}{R}_{OC}\right)$, where *L* is the cavity length, *c* is the light velocity, *L*_*in*_ is the internal cavity loss, and *R*_OC_ is the reflectivity of OC, is estimated to be only < 1 ns, indicating that the resonating signal output circulates only once or twice in the cavity.

A high-Q plane-parallel cavity for the signal was constructed simply by replacing M2 by M3 (high reflectivity (99%) for signal). The intracavity photon lifetime is then extended to ~ 24 ns (corresponding to ~ 50 times cavity roundtrips). Such plane-parallel high-Q cavity, in general, will act as an unstable resonator with no eigenmodes.

In fact, the cavity prevented the lasing of the signal in the vortex mode, thereby resulting in the 3.468 µm vortex idler output with a pair of Y-shaped fringes with ℓ = 1, as shown in Fig. 1i,j. Also, the handedness of the idler output was identical with that of the pump beam. Thus, the cavity forces the OAM of the pump beam to selectively transfer to the idler output. The 1.535 µm signal output then showed a Gaussian spatial form and straight-line fringes without any phase singularities (ℓ = 0), as shown in Fig. 1g,h. It is also noteworthy that the spatial forms of the signal and idler outputs suffered from no degradation at any pump levels.

We now hypothesize that such interesting OAM switching between the signal and idler outputs is determined only by the intracavity photon lifetime, i.e. internal cavity loss, of the resonating signal output. Thus, it might be possible by switching an internal cavity loss with an opto-acoustic modulator (AOM) or a rotating dielectric plate. To understand fully this interesting phenomenon, further investigation is needed by employing additional intracavity elements, such as AOM.

The use of the output couplers with various curvature radii will also enable us to control the cavity mode size, however, we must further appropriately shorten or lengthen the cavity to generate desired mode. The plane-parallel compact cavity allows us to selectively generate a desired vortex or Gaussian mode simply by changing the transmission loss of the output coupler.

**Figure 2:**
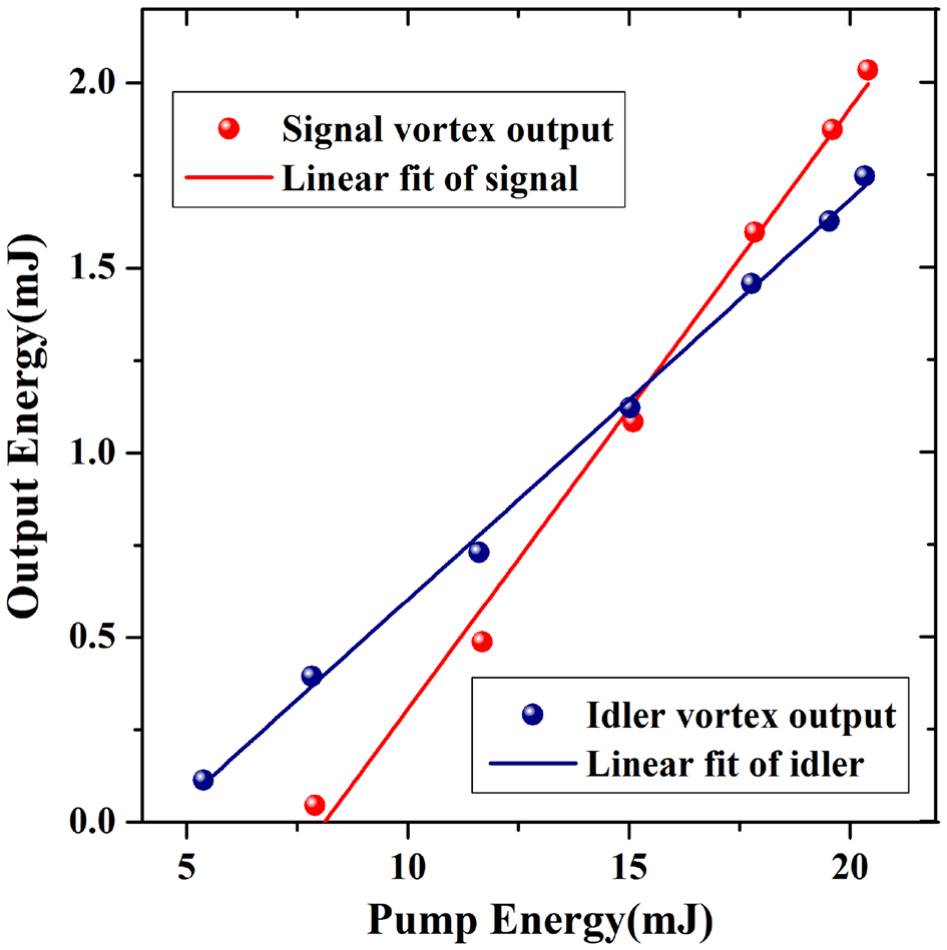
Signal and idler vortex output energy as a function of the pump energy measured for both low- and high-Q cavity configurations.

Figure 2 shows the vortex output energy as a function of the pump energy measured for both low- and high-Q cavity configurations. In the case of a low-Q cavity, a maximum signal vortex output energy was measured to be 2.0 mJ at a maximum pump energy of 21 mJ, corresponding to a slope efficiency of 14%. For the cavity with a high-Q factor, a maximum idler vortex output energy of 1.75 mJ was achieved, corresponding to a slope efficiency of 10% and an oscillating threshold of approximately 4 mJ.

**Figure 3:**
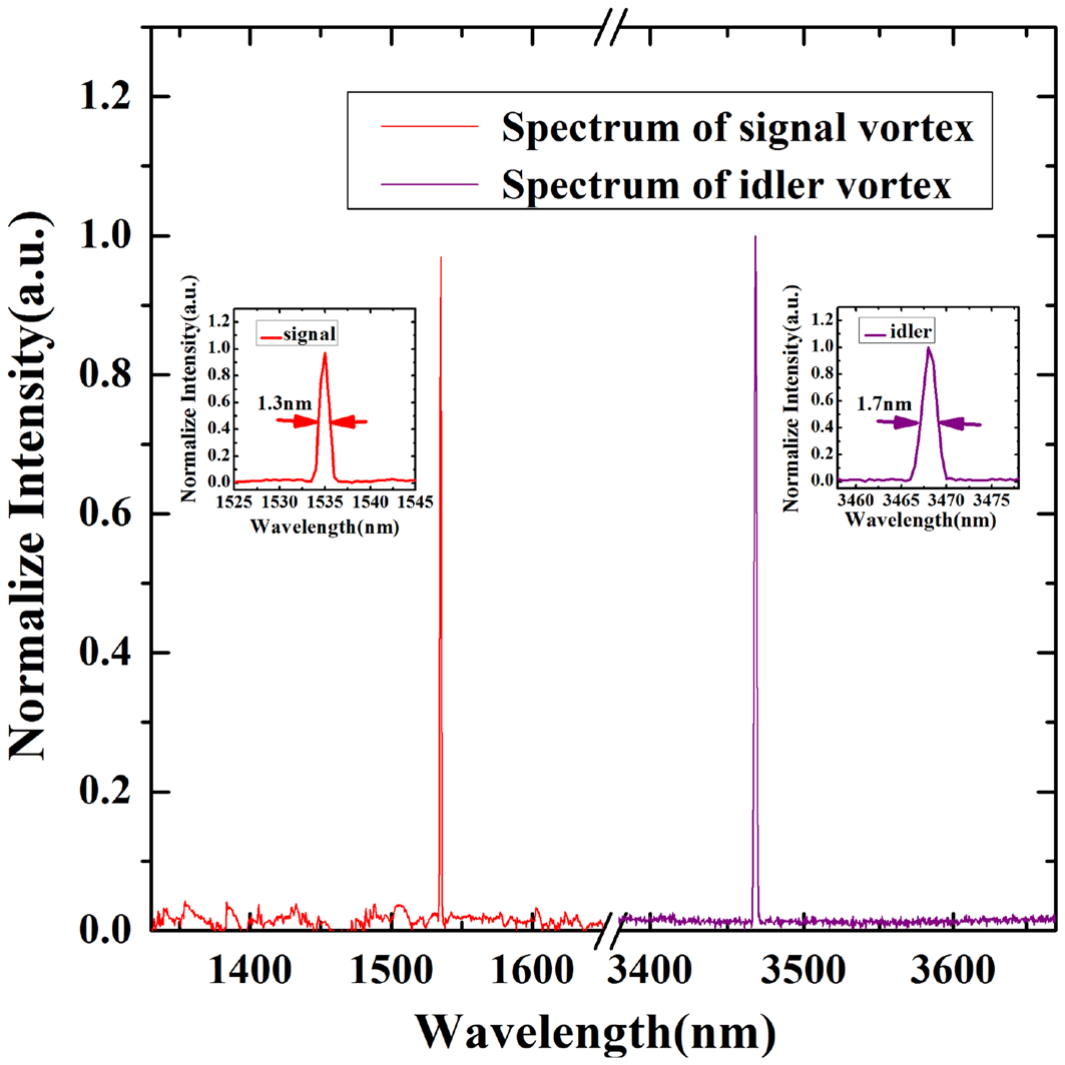
Spectrum of the signal and idler vortex outputs at the wavelength of 1.535 µm and 3.468 µm, respectively. Insets show the spectral bandwidth of the signal and idler vortex outputs.

Figure 3 shows the lasing spectra of the signal and idler vortex outputs measured by using a high performance scanning monochromator (SpectraPro HRS-500, 300 line/mm, aperture size: 50 µm, spectrum resolution: 0.3–0.4 nm in the wavelength range of 1000–5000 nm). The lasing spectral bandwidths of the signal and idler vortex outputs were measured to be *Δλ*_*s*_ ~ 1.3 nm (5.5 cm^−1^) and *Δλ*_*i*_ ~ 1.7 nm (1.4 cm^−1^). It is worth noting that the wavelength of the signal output was close to the cut-off wavelength of our monochromator, thus, the signal output might exhibit such relatively broader linewidth (~ 5.5 cm^−1^). The spectral bandwidth of the idler output was estimated to be 1.4 cm^−1^ at 3.468 µm, and this value was almost similar to those reported in the previous nanosecond OPO works^38^.

Our KTA OPO enables the generation of a high energy, narrow spectral bandwidth, near and mid infrared optical vortex light source, and it will be useful for molecular absorption microscopy with a high spatial resolution beyond the diffraction limit.

## Conclusions

We have demonstrated, for the first time to the best of our knowledge, a milli-joule level, near (1.535 µm) and mid (3.468 µm) infrared optical vortex source formed of a 1-µm vortex pumped KTA OPO with a compact singly resonant linear cavity. At the maximum pump energy of 21 mJ, the maximum signal and idler vortex output energies were measured to be 2.04 mJ and 1.75 mJ, corresponding to slope efficiencies of 14% and 10%, respectively. The spectral bandwidth of the signal and idler vortex outputs were also measured to be Δλ_s_ ~ 1.3 nm (~ 5.5 cm^−1^) and Δλ_i_ ~ 1.7 nm (~ 1.4 cm^−1^), respectively. The non-critical phase-matching crystal was used to avoid the walk-off effects, thereby yielding high quality optical vortex output without any degradation of wavefront.

The wavelengths of signal and idler outputs can be easily tuned only by changing the orientation of the KTA crystal^39^. It is also worth mentioning that the wide wavelength tunability of the signal and idler vortex outputs will be possible by employing cascaded critical phase matching KTA crystals to compensate the walk-off effects. Such wavelength tuanbility of the system will be investigated in the future. The generation of a higher-order optical vortex mode can further be achieved by employing a high-order vortex pumping configuration.

## Experimental method

**Figure 4:**
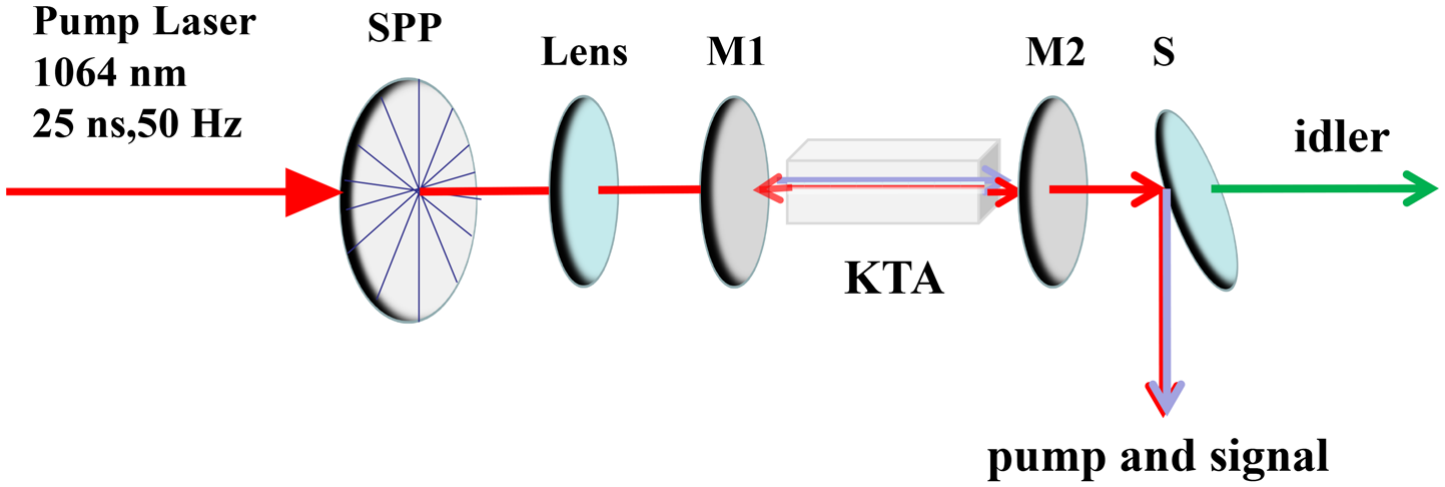
Experimental setup for near and mid infrared KTA optical vortex parametric oscillator.

An experimental arrangement of the KTA OPO is shown in Fig. 4. The pump source used was a conventional Q-switched Nd:YAG laser (Lotis LS-2136; pulse duration: 25 ns; PRF: 50 Hz) radiation at 1.064 µm with a maximum output energy of 21 mJ in a nearly Gaussian spatial mode. Its output was converted into a first-order optical vortex with OAM, *ℓ*, of 1 by employing a spiral phase plate. The pump optical vortex beam was focused to be a 1 mm spot on the KTA crystal by a lens with a focal length of 750 mm. A 30 mm long KTA crystal with an 5 × 5 mm^2^ aperture was used, and it was cut along the x-axis (*θ* = *π*/2, *φ* = 0) to satisfy the type II NCPM among the pump, 1.5 µm signal and 3.5 µm idler outputs. Both faces of the crystal had antireflection (*R* < 0.5%) for the pump, signal, and idler outputs. A plane-parallel single resonant cavity for the signal was formed by using a flat input mirror (M1) with antireflection for 1064 nm and high-reflection (HR) for 1.4–1.6 µm and 3–4 µm, and a flat output mirror (OC). The cavity length was maintained at ca. 50 mm. To swap the OAM of the signal and idler outputs, we used two different OCs, M2 and M3.

A low-Q cavity was formed of M1 and M2 with high reflectivity for the 1.064 µm (pump) beam, partial reflectivity (50%) for 1.4–1.6 µm (signal), and high transmission (> 97%) for the 3–4 µm (idler). A high-Q cavity consisted of M1 and M3 with high reflectivity (99%) for 1.4–1.6 µm (signal) and 1.064 µm (pump) and high transmission (> 97%) for 3.3–4 µm (idler).

The pump beam was observed by a conventional CCD camera. The spatial forms and wavefronts of the signal and idler outputs were measured by using a pyroelectric camera (Spiricon Pyrocam III; spatial resolution: 75 μm). We employed a lateral shear interferometer with a Mach–Zehnder geometry, thereby enabling the optical vortex output to interfere with its own copy with a proper lateral displacement. Individual interferometers were constructed for the signal and idler wavelengths.
